# Differences in Dopaminergic Modulation to Motor Cortical Plasticity between Parkinson's Disease and Multiple System Atrophy

**DOI:** 10.1371/journal.pone.0062515

**Published:** 2013-05-03

**Authors:** Shoji Kawashima, Yoshino Ueki, Tatsuya Mima, Hidenao Fukuyama, Kosei Ojika, Noriyuki Matsukawa

**Affiliations:** 1 Department of Neurology and Neuroscience, Nagoya City University Graduate School of Medical Science, Nagoya, Japan; 2 Human Brain Research Center, Kyoto University Graduate School of Medicine, Kyoto, Japan; University of Toronto, Canada

## Abstract

Dopamine modulates the synaptic plasticity in the primary motor cortex (M1). To evaluate whether the functioning of the cortico-striatal circuit is necessary for this modulation, we applied a paired associative stimulation (PAS) protocol that comprised an electric stimulus to the right median nerve at the wrist and subsequent transcranial magnetic stimulation of the left M1, to 10 patients with Parkinson's disease (PD) and 10 with multiple system atrophy of the parkinsonian type (MSA-P) with and without dopamine replacement therapy (-on/off). To investigate the M1 function, motor-evoked potentials (MEPs) were measured before and after the PAS. In both patient groups without medication, the PAS protocol failed to increase the averaged amplitude of MEPs. The dopamine replacement therapy in PD, but not in MSA-P effectively restored the PAS-induced MEP increase. This suggests that not the existence of dopamine itself but the activation of cortico-striatal circuit might play an important role for cortical plasticity in the human M1.

## Introduction

Dopamine modulates the induction of synaptic plasticity in the striatum and primary motor cortex (M1). Animal models have shown that the dopaminergic signal projecting from the substantia nigra (SN) is essential for inducing cortico-striatal synaptic plasticity in the striatum [1], [2], [3], [4], [5]. Regional and training-specific changes in excitatory synaptic transmission in the striatum have been recorded in brain slices of trained mice [5], while the elimination of dopamine receptors and dopaminergic terminals from the M1 itself specifically impairs the induction of synaptic plasticity and motor skill acquisition [6]. Recently we showed that striatal dopamine release is essential for motor skill learning in humans [7].

Paired associative stimulation (PAS), which combines cortical stimulation by transcranial magnetic stimulation (TMS; through the intra-cortical fibers) and peripheral nerve electrical stimulation (through the thalamo-cortical pathway) with a specific interstimulus interval (ISI), appears to be a powerful method of inducing and evaluating plasticity in the M1 [8], [9], [10]. The effect is thought to involve a similar physiological mechanism to that of associative long-term potentiation (LTP) in animal models [11], [12]. Striatal dopamine depletion caused by degeneration of the nigrostriatal dopaminergic neuron leads to motor disturbances in patients with Parkinson's disease (PD), including symptoms of rigidity, tremor and hypokinesia as well as impairments of motor learning [13], [14], [15], [16]. By applying this method to patients with PD, it was found that M1 cortical plasticity is reduced during off-medication and restored during on-medication [17], [18]. Although M1 motor plasticity is also modulated by dopamine in humans, it is not clear whether this modulation is direct or secondary via the cortico-striatal circuit.

To evaluate this, we applied PAS to patients with multiple system atrophy with predominant parkinsonism (MSA-P) both with and without dopamine-replacement therapy. Recent diagnostic criteria characterize probable MSA-P as a sporadic, progressive, adult-onset disorder including rigorously defined autonomic failure and poorly levodopa-responsive parkinsonism [19]. This poor levodopa response of MSA-P compared with PD is caused by neuropathological differences, involving neuronal loss and gliosis in the substantia nigra as well as the striatum [20], [21].

## Patients and Methods

### Patients

Ten PD patients and 10 MSA-P patients were recruited from Kyoto University Hospital, Kyoto, Japan and Nagoya City University Hospital, Nagoya, Japan. All patients gave their informed written consent to participate, and the study was conducted according to the Declaration of Helsinki and approved by the local ethics committees of the Nagoya city university and Kyoto university institutions, respectively. The TMS exclusive criteria such as use of a pacemaker and medical interference in a patient's body were checked in writing. No patients were receiving antidepressant or neuropsychological medication.

The PD patients were not tremor-dominant and had been responsive to L-dopa therapy for more than two years. MSA-P patients fulfilled established clinical diagnostic criteria after extended clinical follow up at least 3 years. All MSA-P patients were eventually diagnosed as probable MSA of parkinsonian type according to Gilman's criteria [19]. The mean age ± SD of PD patients was 66±7.7 years, while that of MSA-P patients was 59.5±11.2 years. Parkinsonian symptoms in both diseases were assessed using the motor subscale (item 19–31) of the Unified Parkinson's Disease Rating Scale (UPDRS) to provide comparable parameters of motor symptom severity between the two groups. In MSA-P patients, cerebellar dysfunction was examined by gait ataxia, cerebellar dysarthria, limb ataxia, and cerebellar oculomotor dysfunction. Autonomic failure was diagnosed if there was at least one feature of postural hypotension and urinary incontinence.

### Paired Associative Stimulation (PAS)

The optimal motor point for the right abductor pollicis brevis (APB) muscle was identified by connecting a focal TMS (figure-of-eight coil) to the Magstim 200 magnetic nerve stimulator. Surface electromyography (EMG) was recorded from the APB muscle (bandpass, 5–2000 Hz) and the optimal motor point for eliciting the best motor response was established over the M1 45° to the mid-sagittal line. The resting motor threshold (RMT) was defined, in accordance with a previous study [22], as the lowest stimulus intensity required to elicit a motor-evoked potential (MEP) with a peak-to-peak amplitude of >50 µV in the right APB muscle in at least five out of 10 trials. The intensity of the TMS test for following PAS was adjusted to produce an MEP of ∼1 mV from the APB muscle (SI1 mV).

The PAS consisted of a single electrical stimulus delivered to the right median nerve at the wrist (110% of the motor threshold) and a subsequent TMS (with an intensity of SI1 mV) over the left M1. Two hundred and forty pairs of stimuli were delivered at 0.2 Hz for 20 min with an interstimulus interval of 25 ms. To measure the mean peak-to-peak MEP amplitudes, 20 stimuli were delivered over the left M1, both before and immediately after PAS using a stimulus intensity of SI1 mV.

### Experimental Design

All patients participated twice in the PAS study. They were examined in the practically defined off state after the withdrawal of L-dopa/carbidopa and selegiline for at least 12 h and dopamine agonists for at least 24 h (off-medication). To investigate the effect of dopaminergic medication, we repeated the same experiment 2 h after administration of the antiparkinsonian drugs (on-medication) subsequent to at least three days after the off-medication. Peak-to-peak MEP amplitudes for each experiment were measured to determine the motor cortical plasticity (PD-on, PD-off and MSA-P-on, MSA-P-off). To evaluate the somatosensory system, we recorded somatosensory evoked potentials (SEP) from the scalp (Fz and CPc) after right median nerve stimulation (MNS) only during on-medication. The peak latency of N20 was also measured.

### Statistical Analysis

To evaluate the effect of PAS on motor cortex excitability in both patients group, the changes in MEP amplitudes were evaluated using a three-factor analyses of variance (ANOVA), treatment (off or on), time (pre- or post-) and disease (PD or MSA) as the main factors. If necessary, the Greenhouse–Geisser correction was used to adjust for sphericity, changing the degrees of freedom using a correction coefficient epsilon. The Bonferroni correction for multiple comparisons was used for the *post hoc* t-test. The threshold of significance was set at *P*<0.05.

## Results

Patient demographic and clinical details are summarized in Table 1. There was no significant difference among duration of symptoms (2.7±0.7 years vs. 2.7±0.9 years, respectively, *P* = 1.0) at the time of PAS experiment and levodopa-equivalent daily dose between PD and MSA-P patients (230±79 mg vs. 260±70 mg, respectively, *P* = 0.3). There were also no differences in the UPDRS motor scores during the off-medication state (18.4±8.9 vs. 24.5±10.8, respectively, *P* = 0.24). The PD patients showed a higher UPDRS motor score in the off-medication condition than the on-medication condition (18.4±8.9 and 7.8±4.6, respectively, *P = 0.0004 according to *t*-test). The MSA-P patients showed a slightly higher UPDRS motor score in the off-medication condition than in the on-medication condition (24.5±10.8 and 21.9±11.6, respectively, *P* = 0.05). No patients presented with levodopa-induced dyskinesia. Although MSA3 had not yet fulfill the clinical diagnostic criteria at the time of PAS experiment, after 2 years-clinical follow up, this patient presented autonomic failure, cerebellar ataxia and putaminal signal changes in MRI and was eventually diagnosed as probable MSA.

**Table 1:** Patient demographic and clinical details.

Patient	Age	Gender	Duration of	Parkinsonian symptoms		Autonomic	Cerebellar	Babinski sign	MRI singal change or atrophy			Medication
			symptoms	UPDRS (part 3)		failure	dysfucntion	with hyperreflexia	Putamen	Brainstem	Cerebellum	
	(years)		(years)	On	Off							(mg/day)
PD1	63	F	2	3	10	-	-	-	-	-	-	100
PD2	61	F	3	4	14	-	-	-	-	-	-	200
PD3	67	M	2	8	14	-	-	-	-	-	-	100
PD4	68	F	3	6	28	-	-	-	-	-	-	300
PD5	65	F	3	5	17	-	-	-	-	-	-	250
PD6	58	M	4	18	35	+	-	-	-	-	-	300
PD7	75	F	2	6	14	-	-	-	-	-	-	200
PD8	62	M	3	5	11	-	-	-	-	-	-	300
PD9	57	F	3	13	29	-	-	-	-	-	-	250
PD10	62	M	2	10	12	+	-	-	-	-	-	300
MSA1	54	F	3	29	29	+	-	-	+	+	+	300
MSA2	68	F	3	39	39	+	-	-	+	-	-	300
MSA3	42	M	2	7	19	-	-	-	-	-	-	100
MSA4	48	F	2	15	17	+	-	-	+	-	-	300
MSA5	68	M	2	11	13	+	-	-	+	-	-	300
MSA6	75	F	3	13	13	+	-	-	+	+	+	200
MSA7	64	M	3	28	31	+	-	-	+	+	+	300
MSA8	42	F	2	32	34	+	+	-	+	+	+	200
MSA9	76	F	5	34	38	+	+	-	+	+	+	300
MSA10	62	M	2	11	12	+	-	-	+	-	-	300

During off-medication, resting motor thresholds of the maximal stimulator output were 52.9±12.6 % for PD and 57.8±11.0% for MSA patients. During on-medication, these values were 52.9±12.6% and 56.5±11.1%, respectively. There were no significant differences between the two groups, either in on- (*P* = 0.58) or off-medication (*P* = 0.51). SI_1 mV_ off-medication values were 64.2±13.7 % for PD and 68.1±12.6% for MSA, and for on-medication these were 62.9±13.6 % and 68.1±12.6 %, respectively. There were no significant differences between the two groups, either in on- (*P* = 0.43) or off-medication (*P* = 0.46). The strength of MNS at the right wrist was 14.5±1.7 mA for PD and 13.7±1.4 mA for MSA patients during off-medication, and 14.5±2.2 mA and 13.7±1.3 mA, respectively, during on-medication. The onset of N20-P20 did not differ significantly between the two groups for on-medication (19.1±1.0 ms for PD and 19.0±1.2 ms for MSA-P, *P* = 1.0).

**Figure 1:**
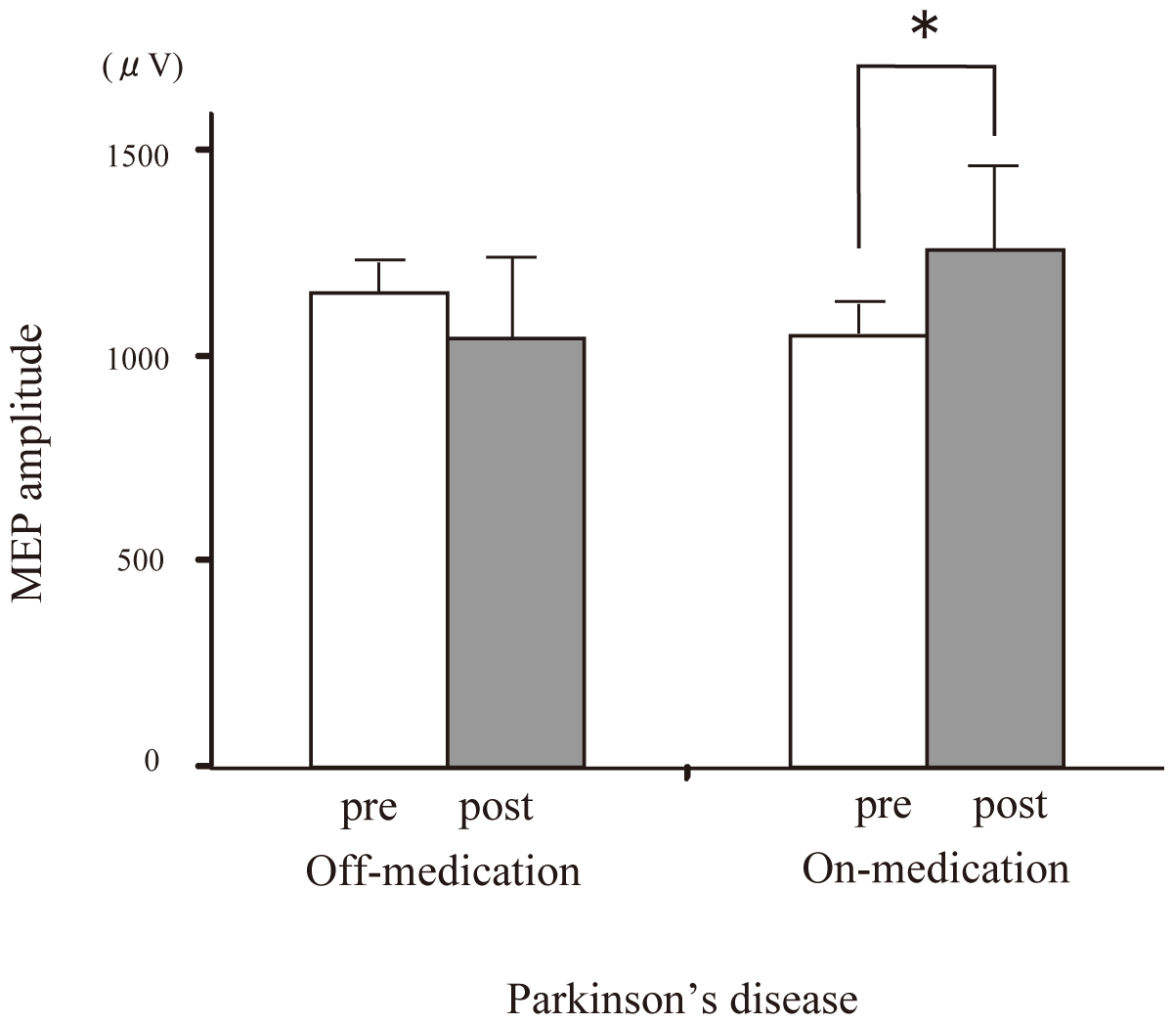
Effect of dopaminergic medication on PAS-induced modulation of the MEP amplitude with PD-on and-off. In PD patients, the average MEP amplitude in the right APB was significantly elevated after dopaminergic medication (*P<0.05).

**Figure 2:**
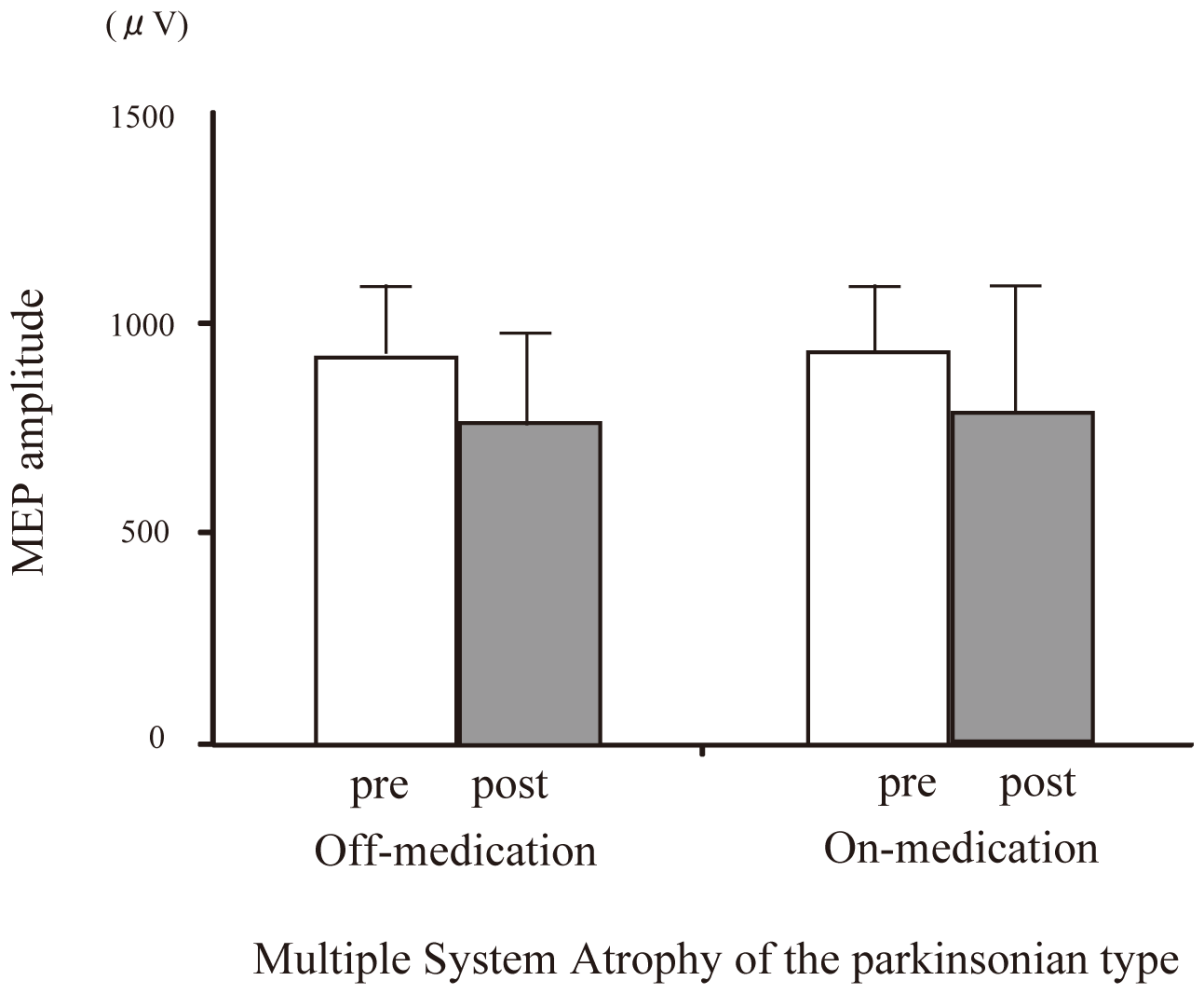
Effect of dopaminergic medication on PAS-induced modulation of the MEP amplitude with MSA-P-on and-off. In MSA-P patients, the average MEP amplitude in the right APB was unchanged by dopaminergic medication.

Looking at the between-subjects effects, the three-factor ANOVA showed no significant main effects for disease (*P* = 0.13), treatment (*P* = 0.20) and time (*P* = 0.36). There were significant time × treatment × disease (*P = 0.006), time × treatment (*P = 0.032), time × disease (*P<0.001) interactions, but not significant disease × treatment interaction (*P* = 0.11). *Post hoc* t-tests revealed the significant increase of MEP amplitude after PAS only for the PD patients with medication (*P = 0.03) (Figure 1). In contrast, there were no significant change of MEP amplitude after PAS in PD patients without medication (*P* = 0.86) and in MSA patients both with (*P* = 0.2) and without medication (*P* = 0.86) (Figure 2).

## Discussion

The main finding of the present study is that the effect of dopamine on cortical plasticity in the M1 differs between PD and MSA-P patients. Although the PAS-induced cortical plasticity in the M1 was decreased in both patients during off-medication, dopamine re-established the plasticity in patients with PD, but not in patients with MSA-P.

The physiological mechanism underlying the reduced M1 cortical plasticity in PD and MSA-P during off-medication appears to be related to degeneration of the nigrostriatal dopamine pathway. In animal studies, the dopaminergic signal projecting from the substantia nigra is essential for inducing the cortico-striatal synaptic plasticity in the striatum [1], [2], [3], [4], [5]. To induce the plastic changes of the cortico-striatal synapse, it is critical to activate the dopamine and NMDA receptors that are situated on the membrane of spiny neurons in the striatum [23], [24], [25]. This co-activation causes the intracellular signal transduction of a common intracellular integrator for inducing the synaptic plasticity in the striatum [3]. Moreover, recent models of the basal ganglia suggest that abnormal pattern of oscillations within the basal ganglia leads to the abnormal motor cortical plasticity via basal ganglia-motor cortical feedback loop [26], [27]. In PD, by applying PAS protocol, it was found that M1 cortical plasticity is reduced during off-medication [17], [18]. Therefore, it is possible that dopamine controls the dynamic circuitry of the cortical plasticity in the M1 through an indirect nigrostriatal pathway via the cortico-striatal circuit in PD and MSA-P.

The administration of dopamine restored the M1 cortical plasticity in PD, whereas cortical plasticity was not restored in MSA-P. These differences in the response to dopamine might be caused by neuropathological differences, possibly involving neuronal loss including medium spiny neuron and gliosis in the striatum [20], [21]. With regard to dopaminergic receptors, by measuring the binding potential of the striatal D2/D3-receptor with ^11^C-raclopride (RAC) positron-emission tomography (PET), loss of D2/D3-receptor binding in the putamen especially the posterior putamen is prominent in MSA-P compared with PD even in the early stage [28] [29]. In the present study, nine out of 10 patients showed putaminal signal changes in MRI indicative of neuronal loss and gliosis in the putamen. Together, these differences in the response to dopamine in PD and MSA-P seem to be caused by dopamine receptors in the putamen. Thus, the striatal dopamine and its receptors are essential for inducing cortical plasticity in the human M1 via the basal ganglia-motor cortical feedback loop.

In the present study, there was no significant difference in disease duration at the time of PAS and levodopa-equivalent daily dose between PD and MSA-P groups. Raclopride-PET study suggested relative and absolute increases in the number of dopamine D2/D3-receptors in the putamen contralateral to the predominant symptoms in the early stage of PD compared with age-matched healthy controls, related to the reduction of presynaptic dopaminergic nerve terminals [30]. However, previous reports showed that the motor cortical plasticity by PAS was not different between PD during on-medication and age-matched healthy controls [17], [18]. Therefore, upregulation of dopamine receptor in the early stage of PD may less modify the MEP facilitation effect by PAS in PD during on-medication.

In animal studies, the background dopamine concentration dependently facilitates LTP in the rat prefrontal cortex through postsynaptic D_1_ and/or D_2_receptor stimulation [31] [32] [33]. LTP induction depends on the level of tonic dopamine stimulation through dopamine receptors, and follows an inverted-U shape curve where both too-low and too-high levels induce LTD rather than LTP [34]. In humans, the modulation of D_2_ receptor activity produces a similar inverted-U shape curve on motor cortical plasticity by using the PAS and transcranial direct current stimulation (tDCS) techniques as a model of bidirectional or non-linear dopamine dose-dependent responses of synaptic plasticity [35], [36], [37], [38]. In the present study, MEP amplitudes tended to be reduced by PAS in on-medications in MSA-P, although this was not statistically significant. Based on these, the differences in dopaminergic modulation of motor cortical plasticity between PD and MSA-P might reflect another model of bidirectional dopamine dose-dependent effects on motor cortical plasticity [38].

To be effective, PAS requires a synchronized TMS pulse and a peripheral sensory input over the M1. A previous SEP study reported that the central sensory conduction time was progressively prolonged in parallel with disease duration in MSA [39]. In this study, since the peak N20 latencies were not prolonged in either PD or MSA-P, the timing of TMS over the M1 appears to be appropriate for inducing cortical plasticity in the M1. Although we did not record the cortical silent period, previous report clearly suggested that the cortical silent period was shortened by PAS in PD during off-medication and prolonged by levodopa treatment in non-dyskinetic group of PD but not in dyskinetic group [17]. Morgante et al. suggested that lack of LTP-like plasticity in the M1 and prolonged cortical silent period by PAS in dyskinetic patients both on-and off-medication contributed to the underlying mechanism of Levodopa-induced dyskinesias [17]. Therefore, the abnormal modification of cortical inhibitory system in M1 by PAS might be also seen in MSA-P.

Taking a clinical point of view, the distinction between PD and MSA-P is sometimes difficult, especially in the early stages of the disease and despite the use of diagnostic criteria [19], [40], [41]. Although neuroradiological methods such as magnetic resonance imaging (MRI) and PET are useful in detecting pathological changes [28], [40], [42], [43], [44], it is still difficult to accurately establish a diagnosis. With regard to TMS, some studies have focused on the motor cortical disinhibition assessed by intracortical inhibition and the cortical silent period and the impaired cortico-spinal tract assessed by the triple stimulation technique [45], [46], [47], whereas others showed that abnormal motor cortical excitability was not correlated with clinical features in MSA [48], [49], [50], [51], [52]. In the present study, one MSA-P patient showed no abnormal putaminal findings at the time of the PAS experiment, though the cortical plasticity in the M1 was already reduced (MSA-P 3 in Table 1). These differences in the cortical plasticity responsiveness to dopamine may provide supportive neurophysiological information in differentiating MSA-P from PD, in addition to neuroimaging findings. However, it would be more important to test motor cortical plasticity in other atypical parkinsonian syndromes such as Progressive Supranuclear Palsy and Cortoco-Basal Degeneration, because it is likely that motor cortical plasticity is altered in these patients. Further study is needed to demonstrate this based on larger patient populations of atypical parkinsonian syndromes and a combination of other quantitative modalities such as MRI and dopamine receptor imaging.
